# Improving causality in microbiome research: can human genetic epidemiology help?

**DOI:** 10.12688/wellcomeopenres.15628.3

**Published:** 2020-04-24

**Authors:** Kaitlin H. Wade, Lindsay J. Hall

**Affiliations:** 1 Medical Research Council Integrative Epidemiology Unit, University of Bristol, Bristol, UK; 2 Population Health Sciences, Bristol Medical School, Faculty of Health Sciences, University of Bristol, Bristol, BS8 2BN, UK; 3 Gut Microbes & Health, Quadram Institute Bioscience, Norwich, NR4 7UQ, UK

**Keywords:** microbiome, human genetics, Mendelian randomization, causality

## Abstract

Evidence supports associations between human gut microbiome variation and multiple health outcomes and diseases. Despite compelling results from *in vivo* and *in vitro* models, few findings have been translated into an understanding of modifiable causal relationships. Furthermore, epidemiological studies have been unconvincing in their ability to offer causal evidence due to their observational nature, where confounding by lifestyle and behavioural factors, reverse causation and bias are important limitations. Whilst randomized controlled trials have made steps towards understanding the causal role played by the gut microbiome in disease, they are expensive and time-consuming. This evidence that has not been translated between model systems impedes opportunities for harnessing the gut microbiome for improving population health. Therefore, there is a need for alternative approaches to interrogate causality in the context of gut microbiome research.

The integration of human genetics within population health sciences have proved successful in facilitating improved causal inference (e.g., with Mendelian randomization [MR] studies) and characterising inherited disease susceptibility. MR is an established method that employs human genetic variation as natural “proxies” for clinically relevant (and ideally modifiable) traits to improve causality in observational associations between those traits and health outcomes. Here, we focus and discuss the utility of MR within the context of human gut microbiome research, review studies that have used this method and consider the strengths, limitations and challenges facing this research. Specifically, we highlight the requirements for careful examination and interpretation of derived causal estimates and host (i.e., human) genetic effects themselves, triangulation across multiple study designs and inter-disciplinary collaborations. Meeting these requirements will help support or challenge causality of the role played by the gut microbiome on human health to develop new, targeted therapies to alleviate disease symptoms to ultimately improve lives and promote good health.

## List of abbreviations

AUC = area under the curve; BMI = body mass index; CAD = coronary artery disease; CKD = chronic kidney disease; FGFP = Flemish Gut Flora Project; GECCO = Genetics and Epidemiology of Colorectal Cancer Consortium; GWAS = genome-wide association study; IBD = inflammatory bowel disease; LD = linkage disequilibrium; MI = myocardial infarction; MR = Mendelian randomization; OR = odds ratio; RCT = randomized controlled trial; RR = risk ratio; SCFA = short chain fatty acid; SD = standard deviation; SNP = single nucleotide polymorphism; TMAO = trimethylamine-N-oxide; T2D = type 2 diabetes

## Introduction

Evidence from microbiome-wide studies has highlighted relationships between the gut microbiome and many complex traits and diseases – from dietary composition, obesity, rheumatoid arthritis and type 2 diabetes (T2D) to Alzheimer’s disease, Parkinson’s disease and depression ^1– 9^. For example, the lower diversity and relative abundances of bacteria within the *Bacteroidetes* vs. *Firmicutes* phyla in obese vs. lean individuals has been observed in studies of mice and humans, with cross-sectional, longitudinal and experimental designs ^10, 11^. Several studies have also demonstrated that the relative bacterial abundances in the *Bacteroidetes* order increases and *Firmicutes* order decreases with low-calorie diets (e.g., through fat or carbohydrate restriction) or surgery-induced weight loss in obese individuals, whereby the gut microbiota composition becomes similar to that of their lean counterparts ^10– 13^. Ostensibly, these studies suggest that the manipulation of the human gut microbiome (e.g., not only through diet and surgery but also via the intake of pre- or pro-biotics, antibiotic usage or faecal microbiome transplants) may have potential as an approach to develop new, targeted therapies and treatments to reduce disease in the population.

However, the design of human studies has been largely observational (with additional potential for experimental biases in sample collection, storage and analysis) and, owing to this, there are the many inconsistences within the literature, casting doubt on the reliability of existing findings. Moreover, causality in these relationships is often difficult to ascertain, with a concerning lack of robust evidence able to discern correlation from causation (despite being called for 14– 16). It is particularly alarming that, despite this lack of evidence, and with much scepticism ^17^, there is a growing market for commercial initiatives targeting the microbiome as a consumer-driven intervention (e.g., ubiome, Viome, BIOHM and Atlas BioMed), where companies ask for, obtain and sequence faecal samples from consumers and prescribe “personalised” nutritional information based on the, often, only sample. Furthermore, there has been an increase in clinical recommendation of pro-biotics – “ *live microorganisms that, when administered in adequate amounts, confer a health benefit on the host*” ^18^ – for treating various diseases or following antibiotic prescription ^19– 21^. However, with the development of such efforts, it is important to recognise that the gut microbiome is a dynamic and complex ecosystem; therefore, careful investigation of the off-target effects of any treatment or intervention intended to alter one or a small number of specific bacteria is required.

Some of the current literature comprising *in vivo* and *in vitro* experiments has provided promising results, which have been supported by small-scale observational studies within humans. However, many studies have failed to be translated between model organisms, and studies within humans have been unconvincing in their ability to provide evidence for causality in these relationships (even those with compelling results from *in vivo* and *in vitro* models). Despite the few examples that have proved successful in their consistency between model organisms and their clinical application in humans (e.g., faecal microbiome transplantation in cases of recurrent *Clostridioides difficile* infection, which has a global success rate of over 80% ^22^), evidence that has not been translated between model organisms impedes any opportunity for harnessing the gut microbiome for reducing the burden of disease in the population and has induced scepticism in its causal relevance in human health ^23, 24^.

Reasons for these discrepancies between and within model organisms include the challenges in the increasing volume of high-dimensional multi-omic data produced and, specifically, how these are integrated using complex bioinformatics and incorporated into traditional study designs, alongside the sensitive experimental models that aim to replicate disease traits in humans. Whilst murine models have played a key role in the emerging gut microbiota research field (given the inability to research all questions within humans), there are important inter-study variations due to experimental design (e.g., sample collection and processing), environmental conditions (i.e., differential microbiome composition between rodent housing facilities), genetic differences and chosen analyses ^25^. Despite some compelling examples exhibiting consistency and providing mechanistic understanding of these relationships within humans and animals (e.g., in malnutrition and obesity ^26, 27^) and between *in silico* and *in vitro* models ^28^, it is still debatable as to whether animal models of the human gut microbiome (and methods used such as germ-free or gnotobiotic mice) are translatable to humans, particularly with all scientific questions ^25, 29^.

In addition to the limitations of *in vitro* and *in vivo* experimental design – currently fuelling the “bottom-up” approach for assessing causality between model systems – there are limitations of the observational human epidemiological study designs. Single-sample observational epidemiological studies (i.e., population samples in cross-section) or case-control studies suffer confounding by lifestyle and behavioural factors, biases (e.g., error in measures of the gut microbiome and non-random or unrepresentative selection of participants) within and between studies and may not be generalizable. Indeed, whilst both murine and human studies have provided support for a relationship between the gut microbiome and diseases such as inflammatory bowel diseases (IBDs) and T2D ^10, 30^, these study designs usually include the assessment of differential gut microbiome compositions within clinical patients (i.e., those who already have the disease of interest) compared to controls. Meta-analyses of studies like these can provide some insight into consistency and robustness of potential findings, which may lead to greater precision of observed associations (e.g., with metagenomic signatures within colorectal cancer cases ^31, 32^), but the direction of causality in these relationships is ambiguous. Specifically, is it that differences in the gut microbiome protect/exacerbate a disease or is it the disease state itself that is leading to variation in the gut microbiome composition? Furthermore, if evidence of a likely causal effect of a component of the gut microbiome on the risk of a disease is provided, this does not imply that the same is true for the progression of that disease (and vice versa). Specifically, treating individuals who suffer from, say, IBD with a pre-/pro-biotic that promotes/contains bacteria found to be associated with the risk of developing IBD may not lead to better prognosis of IBD after diagnosis.

Given that the gut microbiome is a dynamic system, assessment of the prospective changes in this system alongside the development of disease and variation in health outcomes is required. Longitudinal cohort studies have developed some understanding in the transitional association of the gut microbiome and specific health outcomes over a relatively short period of time (e.g., with childhood obesity, type 1 diabetes and adult weight gain ^33– 36^). However, these are still limited by traditional observational epidemiological complications (i.e., confounding and bias) if not designed or conducted well and, usually, by statistical power given usually small sample sizes.

Randomized controlled trials (RCTs) in this area have made some steps towards understanding the causal role played by the gut microbiome in disease. However, many mainly focus on alleviating symptoms within patients who have an established condition ^37– 39^. RCTs have focused on the influence of diet, pre- and pro-biotics, or antibiotics on the gut microbiome and related traits and have presented an array of conclusions ranging from a preventative to detrimental role of these interventions. However, most of these RCTs consist of fewer than 50 participants, who tend to be selected based on disease status and who are often on different medications, which are difficult to control ^40^. Only a handful of registered trials to date have completed with tangible results and more have been terminated, suspended or withdrawn ^40^. Given the current literature, trials focusing on disease prevention in healthy individuals or those understanding how variations within the gut microbiome can promote good health are imperative. Whilst larger efforts are ongoing (e.g., as of July 2019, there were approximately 650 RCTs of the gut microbiome still recruiting), such trials are likely not feasible to answer every scientific question and are importantly expensive, time-consuming and sometimes unethical, particularly within a healthy human population. The application of alternative causal inference methods in this context are needed to improve causal inference and help elucidate the role played by the gut microbiome in human health and disease.

## MR

**Figure 1.**
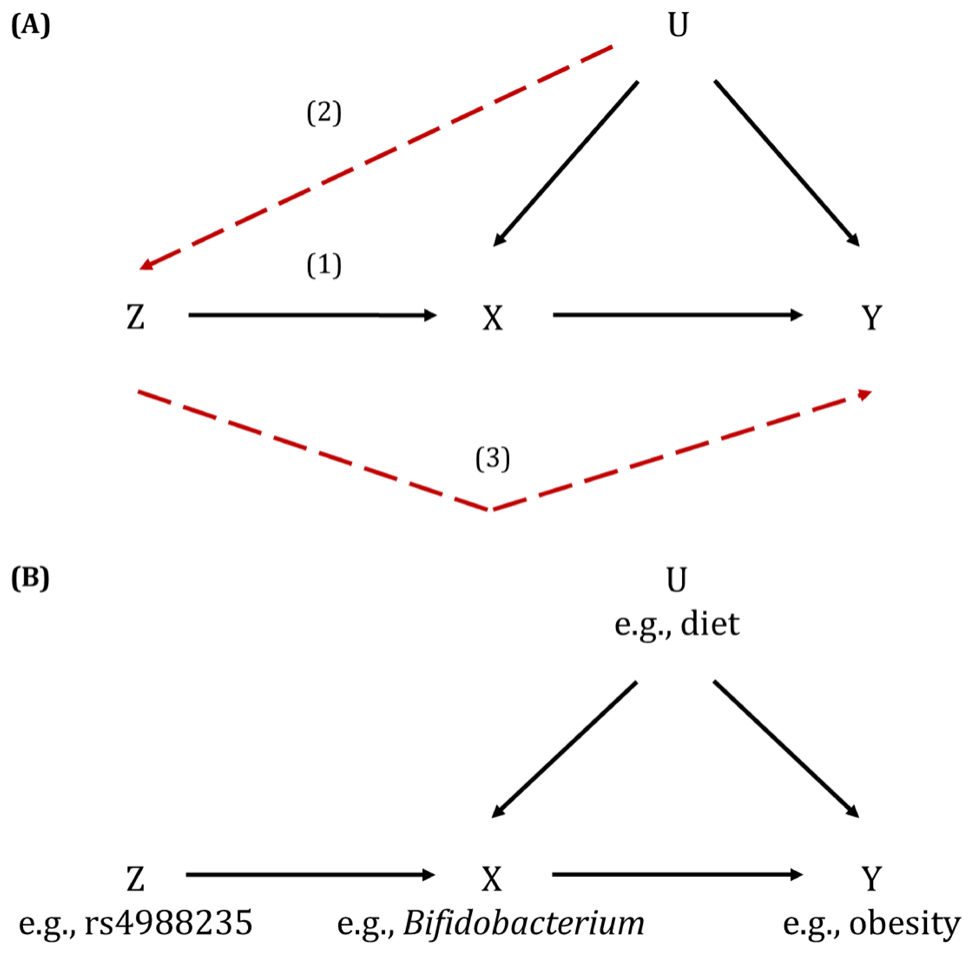
Framework, assumptions and example of Mendelian randomization (MR) in the context of gut microbiome research. ( **A**) MR relies on the following three core assumptions: (1) the genetic variant(s) being used as an instrument (Z) is associated with the exposure (X); (2) the genetic variant(s) are independent of measured and unmeasured confounders (U) of the association between the exposure (X) and outcome (Y); and (3) there is no independent pathway between the genetic variant(s) and outcome (Y) other than through the exposure (X) – known as horizontal pleiotropy or the exclusion restriction criteria. ( **B**) Example of MR applied to understanding the causal role played by Bifidobacterium and obesity using the rs4988235 SNP (i.e., the lactase persistence genetic variant within the *MCM6* locus) as an instrument (see text for discussion).

MR is an approach that uses human genetic variation (usually single nucleotide polymorphisms [SNPs], identified in genome-wide association studies [GWASs]) to act as a “proxy” measure for exposures of interest (e.g., here, the gut microbiome) ^41– 43^. Provided a number of key assumptions are met ( Figure 1), these genetic variants can be argued to have properties that approximate those of “instruments” and thus can be used to estimate the causal effect of a trait on disease or health outcome ^44^. Theoretically analogous to arms of an RCT, genetic variants used in MR are largely independent of confounding factors, due to the random nature of their allocation within a population in the absence of any population stratification. These genetic variants are also not modified by the later development of disease or health outcome and, with very accurate genotyping being commonplace, measurement error is largely reduced. Therefore, at a population level, the portion of variance in the modifiable trait explained by human genetic variants (unlike the direct measurement of the trait itself) can be used to model situations that are free of the aforementioned limitations that would otherwise weaken causal inference in observational studies.

MR expands upon traditional genetic analyses (such as candidate gene studies, GWAS or aggregating genome-wide variation to inform characterisation of disease susceptibility) by harnessing randomly allocated genetic variation identified as being associated with variation in a particular trait to specifically interrogate the causal implications of that trait on a disease or health outcome, in a manner comparable to RCTs. Such analyses can be done at a fraction of the time and cost required for a large-scale RCT, by exploiting data from pre-existing and large-scale genetic studies, and can be used to ask many of the scientific questions that may not be feasible or ethical with RCTs ^41, 42^. Particularly demonstrative of the advantages of the MR paradigm is the example of selenium and prostate cancer ( Box 1).

**Box 1. Illustration of Mendelian randomization in conjunction with randomized controlled trials – the SELECT trial**

Since the early 2000s, many observational studies have implicated the protective role of selenium supplementation or intake (usually determined by measured toenail selenium levels) on both overall and advanced prostate cancer risk ^45, 46^. These findings motivated the initiation of the prospective, double-blinded Selenium and Vitamin E Cancer Prevention Trial (SELECT) ^47^, which randomized the supplementation of selenium, along with other antioxidants, by oral dose to understand its possible causal role on prostate cancer. Despite initial compelling results from observational studies, and after costing $114 million to initiate and conduct, SELECT was terminated prematurely (after 7 years, as opposed to the 12 planned years), as the initial trial results implicated little evidence supporting a protective role of higher selenium levels on the risk of prostate cancer (hazard ratio (HR): 1.09; 99% confidence interval (CI): 0.93-1.27). There was also some evidence to support a detrimental role of selenium on advance prostate cancer (HR: 1.21; 99% CI: 0.90-1.63) and a potentially off-target, detrimental effect of selenium supplementation on T2D within 5 years of the trial (relative risk (RR): 1.07; 99% CI: 0.94-1.22). More recently, complementary MR analyses using 11 SNPs as instruments for circulating selenium levels similarly provided little evidence of a protective role on prostate cancer risk (odds ratio (OR): 1.01; 95% CI: 0.89-1.13) and some evidence supporting the detrimental impact on the risk of advanced prostate cancer (OR: 1.21; 95% CI: 0.98-1.49) and T2D (OR: 1.18; 95% CI: 0.97-1.43), consistent with the trial but in a fraction of the time and with effectively no cost ^48^.

Given the inconsistencies between studies aimed at estimating the causal role of the gut microbiome in human health and disease, MR provides the opportunity to assess causality in observed associations not only between the gut microbiome and health outcomes but also the impact of various traits on the gut microbiome itself, without the need for costly RCTs or lab-based study designs in the first instance. Results derived from the application of MR in this context also provides a potential mechanism to direct the prioritisation of characteristics of the gut microbiome as interventional targets (e.g., via dietary regulation or using pre- and pro-biotics), to inform clinical and public health guidelines and to improve population health in an efficient and cost-effective manner. For a full description and definitions of terminology, methods and assumptions specific to MR, please see the online MR Dictionary ^43^.

## Current applications of MR

Our understanding of the host (i.e., human) genetic contribution to the gut microbiome has primarily arisen from candidate gene studies and genome-wide screens in model organisms ^49^. In recent years, and with the advent of higher-throughput technologies that are able to capture measurements of the gut microbiome at scale, several GWASs in humans have been conducted to further uncover host genetic variation that shapes the gut microbiome ^50– 57^. Together, these initial GWASs have identified associations between more than 100 human genetic variants associated with constituents of the gut microbiome (e.g., microbial diversity, taxon abundance and community structure). However, there has been limited overlap of identified host genetic variants implicated as being associated with the gut microbiome across studies. With the existence of host genetic variants associated with the gut microbiome, we and others have applied MR to appraise causality in the relationships between the gut microbiome and human health ^58– 61^. However, it is of upmost importance for careful examination and interpretation of MR-derived causal estimates, host genetic effects and the assessment of other benefits that the integration of human genetics to this field may provide in appraising causality. The current applications of MR in microbiome research are described below, with pertinent limitations shared between these applications discussed.

### Microbiota genera and ischemic heart disease, T2D and risk factors

In 2018, Yang *et al*. used MR to assess the causal effect of 27 component genera of the gut microbiome on ischemic heart disease, T2D, adiposity, lipid levels and insulin resistance using human genetic variation that had previously been associated with these particular bacterial taxa measured with 16S rRNA sequencing ^58^. In this case, host SNPs (i.e., human genetic variation) associated with these 27 genera were obtained from previous studies ^51– 53, 55, 57^, with highly correlated SNPs removed based on linkage disequilibrium (LD; based on an r ^2^ ≥ 0.8). These were also crossed-referenced with Ensembl and the GWAS catalog to remove potentially pleiotropic SNPs (i.e., those that may have an effect on the outcome other than through the exposure of interest) and to reduce possibility of invalidating the third MR assumption ( Figure 1).

By using MR to analyse the causal impact of these 27 genera on cardiometabolic disease and related traits, authors found evidence that a greater relative abundance of bacteria in the *Bifidobacterium* genus was associated with a 1.5% lower odds of ischemic heart disease (odds ratio [OR]: 0.99; 95% CI: 0.97-1.00), a 0.01 standard deviation (SD) lower body mass index (BMI; 95% CI: 0.01-0.02) and a 0.03 SD lower level of lipoprotein cholesterol (95% CI: 0.02-0.03).

This application of MR provides compelling evidence of the causal impact of the gut microbiome on cardiometabolic traits. However, these results were not robust when testing whether the effects of these genetic variants were independent of *Bifidobacterium* (i.e., “horizontal pleiotropy” – Figure 1), suggesting that this bacterial genus may not have been the sole driver of these seemingly beneficial metabolic effects. Additionally, many of the associations between the human genetic variants and the gut microbiome used in the MR analyses were not replicated either within or between the studies from which they were obtained ^51– 53, 55, 57^, questioning the validity of using them in MR analyses, as they may not be reliably associated with the exposure (i.e., invalidating the first MR assumption, Figure 1).

### Short-chain fatty acids and metabolic diseases

In 2019, authors of another study based on a collection of 952 normoglycemic individuals from the LifeLines-DEEP cohort with genetic, metagenomic sequences and faecal short-chain fatty acid (SCFA) levels performed bi-directional MR to assess the association of 245 metagenomic features describing functionality of the gut microbiome (2 of which were linked to SCFA production, 57 unique taxa and 186 pathways) with 17 metabolic and anthropometric traits ^59, 62^. Genetic variants were chosen by conducting a GWAS within 952 individuals from the LifeLines-DEEP cohort. A fairly lenient p-value threshold of 1×10 ^-5^ was used to define host genetic variants independently associated with functional features of the gut microbiome. These genetic variants selected at the lenient threshold were chosen as they explained most variance in the same feature in an independent cohort of 445 individuals, compared to other variants defined using varying thresholds.

Authors found that the microbial functional pathway characterised from metagenomic sequencing involved in 4-aminobutanoate (GABA) degradation (PWY-5022), of which the SCFAs butyrate and acetate are products, was associated with improved insulin response after an oral glucose-tolerance test (characterised by the ratio of the areas under the curve (AUC) for measured insulin and glucose levels, AUC _insulin_/AUC _glucose_). Specifically, by using MR to assess the impact of functionality of the gut microbiome, authors found that each SD increase in the abundance of the PWY-5022 pathway was associated with a 0.16 mU/mmol increase in the AUC _insulin_/AUC _glucose_ (95% CI: 0.08-0.24), which was robust to MR methods that test validity with regards to horizontal pleiotropy. In taxonomic analyses, the bacteria most correlated with the PWY-5022 functional pathway were *Eubacterium rectale* and *Roseburia intestinalis* (both of the Clostridiales order), species known to produce butyrate ^63^. The proposed mechanism explaining these results suggested that host genetic variation influences the gut microbiome composition to modulate GABA degradation, thus, increasing the ability of the pancreas to secrete insulin in response to a glucose challenge.

Whilst the metagenomic features authors used provided more insight into the functionality of the gut microbiome (over and above measuring relative abundances with 16S rRNA sequencing), authors were unable to test the relationship between these features and circulating levels of the SCFAs (e.g., butyrate and propionate), as these were not measured in the study sample. Similar to Yang *et al*., the associations between the genetic variants used in MR analyses and the functional features of the gut microbiome were not replicated in other studies. Additionally, much of the GWAS summary-level data that authors used was adjusted for other covariates (mainly BMI), which may induce false correlations between the exposure and outcome via a certain type of selection bias (i.e., collider bias) and, in the most extreme cases, this can reverse the direction of the causal effect estimate (which was observed in this study) ^64, 65^.

### Microbiota-derived metabolites and cardiometabolic health

A further study conducted by Jia *et al*. used MR to examine the association between the trimethylamine-N-oxide (TMAO) metabolite, produced by processes specific to gut bacteria when metabolising choline from high-fat foods such as eggs and beef, and its predecessors with both continuous measures of cardiometabolic health and diseases ^60^. This study was motivated by the observational epidemiological literature, suggesting that choline, TMAO (a derivative of choline) and carnitine are associated with an increased risk of heart disease and other cardiometabolic diseases, hypothesised through their atherosclerotic effects in blood vessels ^66, 67^. The authors undertook a bi-directional MR analysis to unpick the direction of association of circulating levels of these metabolites with traits relating to adiposity, glycaemic profile, lipids and kidney function alongside diseases including T2D, coronary artery disease (CAD), myocardial infarction (MI), stroke, atrial fibrillation and chronic kidney disease (CKD) ^60^. Genetic variants used in MR analyses as instruments for each of four metabolites (choline, TMAO, carnitine and betaine) were obtained from a GWAS of 217 blood-based metabolites in 2,076 individuals of European descent from the Framingham Heart Study (Offspring Cohort) ^68^ and chosen based on a lenient threshold of “suggestive” genome-wide significance ( *P*<5×10 ^-5^). Given the number of tests being performed, authors set an *a priori* Bonferroni-corrected threshold ( *P*<0.0005) to detect evidence for association.

By using MR to assess the causal role of gut microbiome-derived metabolites and cardiometabolic health, authors found some evidence to suggest that higher circulating levels of choline increased the risk of T2D (OR: 1.84 per 10 units; 95% CI: 1.00, 3.42) and higher circulating levels of betaine reduced the risk of T2D (OR per 10 units: 0.68; 95% CI: 0.48, 0.95). There was little evidence to suggest that any metabolite had a causal role on the continuous measures of cardiometabolic health. In the reverse direction, there was evidence suggesting that a higher liability to T2D may play a causal role in increasing levels of TMAO (0.13 units; 95% CI: 0.06, 0.20). Most of these findings were consistent across multiple MR methods, which test robustness with regards to horizontal pleiotropy (i.e., the third MR assumption, Figure 1).

Results presented by Jia *et al.* provided further insight into the functional relevance ** of the gut microbiome on cardiometabolic disease, performing power calculations and utilising a selection of appropriate sensitivity analyses that appraise validity of derived causal estimates. These results suggested that, by altering levels of these microbiome-dependent metabolites, there may be an opportunity to modify the potential causal impact of the gut microbiome and its metabolic functionality to reduce the risk of T2D. However, it is important to clarify firstly that authors tested the relationship of circulating levels of metabolites with cardiometabolic traits and not characteristics of the gut microbiome itself. Indeed, whilst constituent gut microbiota do produce these metabolites naturally and are associated with their circulating levels, such metabolites can be introduced into the blood stream via other means (e.g., supplementation and diet). Thus, the direct causal impact of gut microbiome in this instance is uncertain and may, in fact, be irrelevant. Similar to the other applications of MR described above, the genetic variants used as instruments for the microbiota-derived metabolites were not replicated in the original GWAS from which they were obtained and were selected based on a fairly lenient threshold (similar to Sanna *et al*. ^58^), questioning the reliability of their use.

### Genome-wide association of gut microbiome variation and causal inference analyses

As is evident from the current applications of MR in the context of microbiome research, one of the main limitations is the limited overlap and replication of identified host genetic variants associated with the gut microbiome across studies. Most recently, a GWAS of the gut microbiome characterised with 16S rRNA sequencing was conducted by Hughes *et al.* in over 3800 individuals from three independent studies, including a discovery sample comprising the Flemish Gut Flora Project and two German replication samples ^61^. Within this analysis, 13 SNPs reached conventional levels of genome-wide significance ( *P*<2.5×10 ^-8^), with some showing low heterogeneity between studies, and two of these reached a strict study-level threshold ( *P*<1.57×10 ^-10^). This novel and persistent collection of SNPs associated with measures of the gut microbiome enabled the application of MR to provide further insight into the causal link between these microbial features and a set of metabolic, inflammatory and neurological traits previously implicated as being associated with the gut microbiome throughout the literature ^61^.

These results provided evidence for causality between five microbial traits and seven outcomes, including evidence for a causal role of bacteria within the *Butyricicoccus* genus on IBDs and bacteria within the *Firmicutes* phylum on waist circumference. The strongest association indicated that presence (vs. absence) of bacteria within the *Dialister* genus decreased the risk of Alzheimer’s disease (risk ratio [RR] with a doubling of the genetic liability to presence vs. absence of *Dialister*: 0.81; 95% CI: 0.73, 0.90). In the reverse direction, there was also evidence for causal relationships between four phenotypes and three microbial traits, including evidence for a causal role of a higher liability to Parkinson’s disease, T2D and Crohn’s disease on bacteria within the *Firmicutes* phylum. The strongest result suggested that individuals with Alzheimer’s disease were more likely to carry bacteria in the *Dialister* genus within their gut (RR for a doubling of the genetic liability to Alzheimer’s disease: 1.81; 95% CI: 1.13-2.89).

Focusing on the components of this latter result, the bi-directional analyses of the causal relationship between bacteria within the *Dialister* genus and Alzheimer’s disease may seemingly be somewhat contradictory (i.e., presence of *Dialister* reducing the risk of Alzheimer’s disease but Alzheimer’s disease presence increasing likelihood of *Dialister*). However, there are two important points to note. Firstly, there may well be a true protective role of the *Dialister* bacteria on the onset of Alzheimer’s disease (hence the inverse effect), which is supported by a recent study proposing this very notion ^69^. Secondly, the single genetic variant used as an instrument in the MR analysis assessing the impact of *Dialister* bacteria on Alzheimer’s disease is within *SORL1*, a gene characterised as being associated with Alzheimer’s disease itself ^70^. Therefore, the question arises as to whether the SNP is indeed a valid instrument for bacteria within the *Dialister* genus (as it may be a pleiotropic SNP, independently associated with Alzheimer’s disease) or whether the mechanism by which the SNP influences Alzheimer’s disease is through its impact on bacteria within the *Dialister* genus. At the present time, is difficult to discern without further biological, functional and mechanistic knowledge. Therefore, there is a requirement for careful examination and interpretation of the host (i.e., human) genetic effects on these microbial traits before using them in such applied analyses.

**Figure 2.**
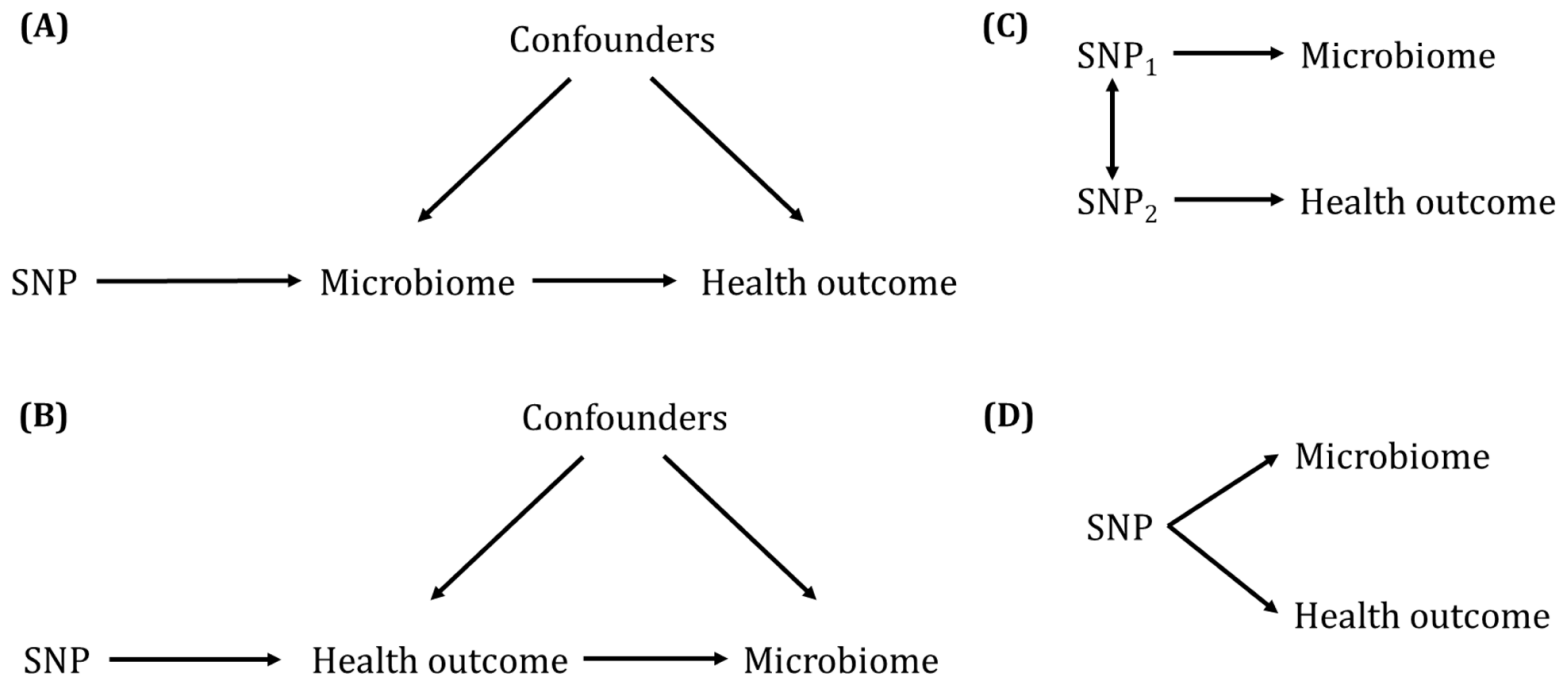
Mechanisms explaining observed associations between genetic variants and the gut microbiome (adapted from Richardson *et al.*) ^71^ testing the association between the gut microbiome and an example health outcome. ( **A**) The genetic variant has an effect on the health outcome, mediated through the microbiome (as in Figure 1) – i.e., the relationship of interest; ( **B**) the genetic variant has an effect on health outcome through other biological mechanisms, which in turn has a downstream effect on the microbiome (i.e., reverse causation); ( **C**) the genetic variant that influences the microbiome is correlated with another genetic variant (i.e., they are in linkage disequilibrium) that influences the health outcome; ( **D**) the genetic variant influences both the microbiome and a health outcome through two independent biological pathways (i.e., horizontal pleiotropy).

One proposed mechanism for examining these complexities and unpicking the link between the human gut microbiome and various health outcomes is utilizing the plethora of human genetic epidemiological methods and sensitivity analyses that specifically explore the validity of host genetic variation in MR analyses. For example, as proposed by Richardson *et al*. ^71^, methods such as colocalization, bivariate genetic fine mapping and bi-directional MR may provide some distinction between reverse causality and either direct or LD-induced horizontal pleiotropy ( Figure 2). As these methods (particularly colocalization and genetic fine mapping) require individual-level and genome-wide information on both the exposure and outcome, these methods will become more feasible with the growing availability of large-scale GWASs of the gut microbiome and other traits.

Indeed, the application of such methods in a recent study provided some insight into the validity of using host genetic variants associated with the human gut microbiome in examining the relationship between gut microbiome variation and colorectal cancer (unpublished). For this, the summary statistics from the 13 SNPs associated with microbial traits derived from 16S rRNA sequencing reported by Hughes *et al.* were combined with those from the Genetics and Epidemiology of Colorectal Cancer Consortium (GECCO) in a two-sample MR analysis ^61^. Results provided evidence that the presence (vs. absence) of uncharacterised bacterial genera within the *Bacteroidales* order increased the risk of colorectal cancer by approximately 8% (95% CI: 2-15%), with no strong evidence that the SNP used as an instrument was associated with other traits or the outcome itself, reducing the likelihood of horizontal pleiotropy.

Whilst these are first steps in the right direction of understanding the utility of host genetic variation in microbiome research for improved causality, there is much room for improvement. Importantly, when appreciating the complexity of these relationships, the integration of human genetics, genetic epidemiological techniques and causal inference methodologies to the field of microbiome research holds great potential.

## Limitations of the MR approach

With the advent of the application of MR within the context of gut microbiome research, and the growing data available for such analyses, there are important limitations and complexities to these applied epidemiological analyses that need to be acknowledged and addressed. The general limitations of MR have been summarised before ^41, 43^, but those most pertinent to microbiome research are currently the lack of robust and reliable genetic variants associated with the gut microbiome and its functionality and the complexity of mechanisms by which host genetic variants impact these microbial traits. This latter complication includes the possibility that identified genetic variants associated with components of the gut microbiome are also associated with the outcome of interest in an MR study through independent mechanisms ( Figure 2). In addition, many of the current studies utilizing MR have used lenient *p*-value thresholds to define the set of included genetic variants, leading to concerns in their instrumental variable quality (via an invalidation of the third MR assumption).

As the pool of increasingly larger-scale GWASs and meta-analyses grow (e.g., most imminently with the MiBioGen initiative ^72^), the number of genetic variants associated with the various characterisations of the microbiome (i.e., bacteria-specific metabolites, bacterial taxa or functional features) will also likely grow. A greater number of genetic variants robustly associated with features of the gut microbiome will further enable the application of the continuously developing plethora of MR methods that require multiple instruments to investigate the effect of confounding, mediation, pleiotropy and invalidation of MR assumptions. However, it seems clear that the environmental contribution to the gut microbiome will be much greater than the host genetic contribution ^8^. At the very least, this calls for greater sample sizes in individual cohort studies and consortia for adequate statistical power within MR analyses testing the causal role of these environmental cues on gut microbiome variation (or, indeed, the role of the gut microbiome on disease). It is important to note that the mechanisms by which these genetic variants are associated with the gut microbiome need to be carefully considered and will rely on comprehensive functional and biological experiments and knowledge from both animal and human models that will only be possible with inter-disciplinary collaboration. Furthermore, the application of MR will only be relevant to the components of the gut microbiome that are detectibly heritable and, where this is not the case, alternative approaches to interrogate causality in relationships between the gut microbiome and health traits will be required.

Understanding the complex mechanisms that link genetic variation with the gut microbiome will be particularly important when interpreting results obtained from MR analyses ( Figure 2). As an example of this, in the GWAS conducted by Hughes *et al.* ^61^, bacteria in the *Bifidobacterium* genus were associated with the well-characterized rs4988235 lactase persistence variant at the *MCM6* locus, which is common in European populations and the only persistent signal among existing microbiome-wide GWASs. Each additional copy of the lactase persistent allele decreases the relative bacterial abundance of *Bifidobacterium*, where individuals predisposed to be lactose tolerant are likely to have a reduced average *Bifidobacterium* bacterial abundance within their gut. This observation is supported biologically as species and strains of *Bifidobacterium* can metabolise lactose (preferentially over other simple sugars), where individuals who are lactose intolerant have higher levels of *Bifidobacterium* to aid digestion, and where supplementation of *Bifidobacterium* has been shown to reduce lactose intolerance in humans ^73, 74^. In this study, by using the rs4988235 variant as an instrument in MR analyses to interrogate the causal role of bacteria in the *Bifidobacterium* genus in the aetiology of the collection of metabolic, inflammatory and neurological traits, there was suggestive evidence of causal effects with adiposity-related traits. Specifically, a greater relative bacterial abundance of *Bifidobacterium* had a potentially causal role in lowering waist circumference, BMI and waist-hip ratio (where the association with BMI was consistent with that described by Yang *et al*. ^58^).

However, it is currently difficult to determine whether this relationship with adiposity-related traits is the direct product of variations in the relative abundance of *Bifidobacterium* (i.e., via immune modulation or the production of SCFAs, for example ^75, 76^) or the direct impact of rs4988235 variation on adiposity via other exposures such as dietary composition (e.g., milk intake), independently of *Bifidobacterium* (i.e., horizontal pleiotropy – Figure 2D). Therefore, whilst there may appear to be a causal effect of *Bifidobacterium* on adiposity measures, this observation may be an artefact resulting in the independent impact of the rs4988235 on both *Bifidobacterium* and adiposity, which is difficult to discern without further functional knowledge of the host genetic variants being used as instruments for the gut microbiome in MR analyses. This ambiguity is particularly pertinent at a time where there are few genetic variants reliably associated with characteristics of the gut microbiome.

In addition to these MR-specific limitations, it is worth revisiting the general limitations with microbiome research that have likely driven the limited overlap of genetic variants consistently associated with gut microbiota between microbiome-wide GWASs. These include (but are certainly not limited to) differences in protocols/standards for sample collection and storage, DNA extraction method (including chosen hypervariable region for PCR and sequencing methods ^77^), PCR primers, and amplicon vs. shotgun sequencing ^78, 79^. Whilst 16S rRNA amplicon sequencing is useful in providing insight into the types of bacteria present within samples, one particular issue in current studies is the limited resolution afforded by this technology. However, as studies using 16S sequencing combine for undertaking harmonized GWASs (e.g., with the MiBioGen initiative), the power afforded by larger sample sizes will be invaluable for understanding the host genetic contribution to the gut microbiome. This, in combination with future studies using data from complementary technologies (e.g., shotgun metagenomics achieving strain-level resolution, metatranscriptomics, proteomics and metabolomics) able to provide more refined measures of components and functionality of the gut microbiome, will enable a more comprehensive understanding of the role played by these microorganisms in health and disease and the mechanisms by which these occur. In addition, whilst most studies have focused on the bacterial component of the gut microbiome, it is important to interrogate the causal role that other integral microorganisms (i.e., fungi, viruses and archaea) play in the development and progression of host disease and health outcomes over the lifecourse.

## Conclusions

MR is an established approach that uses human genetic variation to estimate causal associations in observational epidemiological relationships and can be used to provide further insight into the causal relevance of the gut microbiome in human health and disease. The applications discussed here currently flag the potential of MR analyses following the growing collection of genetic association data for the human gut microbiome, but there are also important issues likely to arise with a naïve integration of complex GWAS results to understanding causes of health outcomes without in-depth knowledge of the host genetic variants themselves and the performance and pitfalls of MR methodology ^41, 80^. Whilst MR was motivated to confer certain advantages over traditional epidemiological study designs, like any study design within epidemiology, it is not exclusively adequate to conclusively demonstrate causality. Therefore, there is a continued need for triangulation across multiple traditional epidemiological approaches and inter-disciplinary collaboration to support or challenge causality of the role played by the gut microbiome on human health and to understand the mechanisms by which these relationships occur. Such partnerships are necessary to maximise translation into the development of new, targeted therapies to alleviate disease symptoms to ultimately improve lives, and promote good health whilst preventing ill health ^81^.

## Data availability

No data are associated with this article.
