# Supplementary material for: Improving causality in microbiome research: can human genetic epidemiology help?
Source: Wellcome Open Res. 2020 Apr 24;4:199. Originally published 2019 Dec 10. [Version 3] doi: 10.12688/wellcomeopenres.15628.3 (PMC7217228; doi:10.12688/wellcomeopenres.15628.3)
Supplement: Peer review history

# Peer review: Reviewer response for version 3

**DOI:** 10.21956/wellcomeopenres.17426.r38582

**Published:** 2020-05-11

**Authors:** Kiyoshi Takeda

**Affiliations:** 1 Laboratory of Immune Regulation, Department of Microbiology and Immunology, Graduate School of Medicine, WPI Immunology Frontier Research Center, Osaka University, Osaka, Japan

In this review article, the authors discuss how studies on microbiome can be translated into human health & diseases, particularly by focusing on the utility of Mendelian randomization (MR) studies. The manuscript, describing usefulness and limitation of MR studies, is well written.

 It seems that the authors describe the advantage of MR studies based on the concept that microbiota composition is influenced by the host genetics. However, environmental factors, particularly dietary habits, rather than genetic factors, have a great impact on microbiome. It would be better to clearly mention this point. Given that a limited fraction of microbiota is influenced by the host genetics, it would also be important to consider the possibility for application of MR studies to link the microbiota (influenced by environmental factors) and the human diseases (caused by genetic factors).

Is the review written in accessible language?

Yes

Are all factual statements correct and adequately supported by citations?

Yes

Are the conclusions drawn appropriate in the context of the current research literature?

Yes

Is the topic of the review discussed comprehensively in the context of the current literature?

Yes

Reviewer Expertise:

mucosal immunology

I confirm that I have read this submission and believe that I have an appropriate level of expertise to confirm that it is of an acceptable scientific standard.

# Peer review: Reviewer response for version 3

**DOI:** 10.21956/wellcomeopenres.17426.r38517

**Published:** 2020-04-24

**Authors:** Serena Sanna

**Affiliations:** 1 Department of Genetics, University of Groningen, Groningen, The Netherlands; 2 Institute of Genetic and Biomedical Research (IRGB), Consiglio Nazionale delle Ricerche (CNR), Monserrato, Italy

Thank you, I do not have any further comment.

Is the review written in accessible language?

Yes

Are all factual statements correct and adequately supported by citations?

Partly

Are the conclusions drawn appropriate in the context of the current research literature?

Yes

Is the topic of the review discussed comprehensively in the context of the current literature?

Yes

Reviewer Expertise:

Genetic mapping, Statistical Genetics, Mendelian randomisation methods, population based microbiome studies.

I confirm that I have read this submission and believe that I have an appropriate level of expertise to confirm that it is of an acceptable scientific standard.

# Peer review: Reviewer response for version 2

**DOI:** 10.21956/wellcomeopenres.17347.r38333

**Published:** 2020-04-21

**Authors:** Serena Sanna

**Affiliations:** 1 Department of Genetics, University of Groningen, Groningen, The Netherlands; 2 Institute of Genetic and Biomedical Research (IRGB), Consiglio Nazionale delle Ricerche (CNR), Monserrato, Italy

I thank the authors for incorporating my suggestions. 

 I only want to get back again at point 4, because I think the explanation given on the study doesn't fully reflect the original design. I suggest the following:

*“Genetic variants were chosen by conducting a GWAS within 952 individuals from the LifeLines-DEEP cohort. A fairly lenient p-value threshold of 1x10-5 was used to define host genetic variants independently associated with functional features of the gut microbiome. Genetic variants selected at this threshold explained most variance of the same feature in an independent cohort of 445 individuals, compared to variants selected with different thresholds”*

Is the review written in accessible language?

Yes

Are all factual statements correct and adequately supported by citations?

Partly

Are the conclusions drawn appropriate in the context of the current research literature?

Yes

Is the topic of the review discussed comprehensively in the context of the current literature?

Yes

Reviewer Expertise:

Genetic mapping, Statistical Genetics, Mendelian randomisation methods, population based microbiome studies.

I confirm that I have read this submission and believe that I have an appropriate level of expertise to confirm that it is of an acceptable scientific standard.

# Response

**Published:** 2020-04-21

**Authors:** Kaitlin Wade

**Affiliations:** University of Bristol, UK

We have incorporated the changes, as requested by the Reviewer.

# Peer review: Reviewer response for version 1

**DOI:** 10.21956/wellcomeopenres.17122.r37954

**Published:** 2020-03-13

**Authors:** Serena Sanna

**Affiliations:** 1 Department of Genetics, University of Groningen, Groningen, The Netherlands; 2 Institute of Genetic and Biomedical Research (IRGB), Consiglio Nazionale delle Ricerche (CNR), Monserrato, Italy

This review is clearly written and comprehensive. It explains the urgent need in identifying causal relationships between microbiome and diseases before moving to real clinical application and to commercial kits, and then introduces the Mendelian Randomization approach that has been recently used in this context. The authors present findings and limitations of these studies, and also more in general the *pros *and *cons *of Mendelian Randomization in application to microbiome. 

 I have few minor suggestions for further improvement:

- Introduction. When discussing the potential role of microbiome modulation as therapy, I think it is important to notice that despite causality can be proved (either by MR or mechanistic studies in mice/humans), we have to 1) remember that gut microbiome is an ecosystem and thus we need to also investigate carefully the side effects of any type intervention intended to module one specific bacteria and 2) remember that evidence for causal relationship with a disease doesn’t necessarily mean that the scenario is reversible, in other words, that modulating that specific bacteria/function will not necessarily cure the disease (while remains a valuable information in terms of prevention).
- MR paragraph. Please remark that MR can be run using limited time and reduced costs **because **it takes advantage of pre-existing large scale genetic studies (otherwise the cost and time are not that limited, even if still less than many RCT).
- MR paragraph – limitations. Please add a note that MR is a great source but only applicable to those bacteria that are modulated by genetic of the host – only a small fraction of bacteria are, so alternative approaches to detect causality are still much needed. 
- Page 6, please amend the details of the study by Sanna *et al.* (2019). In this study, the number of individuals used to carry out the GWAS was 952. An additional independent cohort of 445 individuals was used to derive the optimal threshold for selecting the instruments; specifically this was chosen as the threshold that selected the set of independent variants that, when used in a polygenic risk score in the independent cohort, explained the most of the variance of the same microbiome feature. *[This point explains my answer “partly”  to the question “Are all factual statements correct and adequately supported by citations?”]*
- Page 7 second paragraph. Please add the note that some of the complimentary approaches to inform MR (for example the bivariate finemapping utilized by Richardson *et al.*) – require individual level data of both the exposure and the outcome, and thus are currently impractical to apply to diseases, as there are not such large scale studies of microbiome on case-control studies.
- Page 2, last sentence of the second paragraph. I suggest to add a sentence remarking that to solve this ambiguity, large studies are extremely needed as they will likely lead to multiple associated SNPs, which will help MR to better investigate effect of confounders.

Is the review written in accessible language?

Yes

Are all factual statements correct and adequately supported by citations?

Partly

Are the conclusions drawn appropriate in the context of the current research literature?

Yes

Is the topic of the review discussed comprehensively in the context of the current literature?

Yes

Reviewer Expertise:

Genetic mapping, Statistical Genetics, Mendelian randomisation methods, population based microbiome studies.

I confirm that I have read this submission and believe that I have an appropriate level of expertise to confirm that it is of an acceptable scientific standard.

# Response

**Published:** 2020-03-24

**Authors:** Kaitlin Wade

**Affiliations:** University of Bristol, UK

We wanted to thank the Reviewer for their kind words on the writing and understanding of the importance of this area. We hope that this report provides understandable insight into the Mendelian randomization approach and how this may help further integrate epidemiology and microbiology and interrogate causal relationships between the microbiome and health outcomes. We also thank the Reviewer on their suggestions for improvement, which we have addressed below. For each comment made the Reviewer (in bold) we have responded and added the italicized excerpt. 

**1. Introduction. When discussing the potential role of microbiome modulation as therapy, I think it is important to notice that despite causality can be proved (either by MR or mechanistic studies in mice/humans), we have to 1) remember that gut microbiome is an ecosystem and thus we need to also investigate carefully the side effects of any type intervention intended to module one specific bacteria and 2) remember that evidence for causal relationship with a disease doesn’t necessarily mean that the scenario is reversible, in other words, that modulating that specific bacteria/function will not necessarily cure the disease (while remains a valuable information in terms of prevention).**

The Reviewer makes an excellent point regarding the complexity of the gut microbiome and the implication of this complexity within possible therapies and interventions. We have added a sentence to the introduction to incorporate this point, as follows:

*“However, with the development of such efforts, it is important to recognise that the gut microbiome is a dynamic and complex ecosystem; therefore, careful investigation of the off-target effects of any treatment or intervention intended to alter one or a small number of specific bacteria is required.”*

Additionally, the Reviewer’s point regarding implications of findings to disease prognosis is valuable and, indeed, an important consideration when interpreting findings from all epidemiological studies focusing on disease risk in comparison to disease prognosis. We totally agree that, even if evidence points to a causal impact of the gut microbiome on the risk of a disease, this certainly does not imply that the same is true for the progression of that disease (i.e., treating individuals who suffer from a disease with, say, a pre-/pro-biotic that promotes/contains bacteria associated with the risk of that disease may not lead to a better prognosis – or, as the Reviewer states, cure that disease). This is an important distinction to make and is only resolved by specifically assessing the association between the gut microbiome and disease progression, prognosis or survival (rather than disease risk). As it stands, the application of Mendelian randomization to the study of disease progression is in its infancy, with methods being developed to address the additional caveats of studying populations selected on case-only status, as outlined by Paternoster et al. (e.g., that proposed by Dudbridge et al. and the Slope-Hunter method proposed by Mahmoud et al.). To address this comment, we have stated the following to emphasize this point.

*“Furthermore, if evidence of a likely causal effect of a component of the gut microbiome on the risk of a disease is provided, this does not imply that the same is true for the progression of that disease (and vice versa). Specifically, treating individuals who suffer from, say, IBD with a pre-/pro-biotic that promotes/contains bacteria found to be associated with the risk of developing IBD may not lead to better prognosis of IBD after diagnosis.”*

**2. MR paragraph. Please remark that MR can be run using limited time and reduced costs because it takes advantage of pre-existing large scale genetic studies (otherwise the cost and time are not that limited, even if still less than many RCT).** 

We have edited a sentence in the MR paragraph to clarify this point:

*“Such analyses can be done at a fraction of the time and cost required for a large-scale RCT, by exploiting data from pre-existing and large-scale genetic studies, and can be used to ask many of the scientific questions that may not be feasible or ethical with RCTs[45, 46].”*

**3. MR paragraph – limitations. Please add a note that MR is a great source but only applicable to those bacteria that are modulated by genetic of the host – only a small fraction of bacteria are, so alternative approaches to detect causality are still much needed. ** 

We have added the following sentence to the appropriate section:

*“Furthermore, the application of MR will only be relevant to the components of the gut microbiome that are detectibly heritable and, where this is not the case, alternative approaches to interrogate causality in relationships between the gut microbiome and health traits will be required.”*

**4. Page 6, please amend the details of the study by Sanna et al. (2019). In this study, the number of individuals used to carry out the GWAS was 952. An additional independent cohort of 445 individuals was used to derive the optimal threshold for selecting the instruments; specifically this was chosen as the threshold that selected the set of independent variants that, when used in a polygenic risk score in the independent cohort, explained the most of the variance of the same microbiome feature. [This point explains my answer “partly” to the question “Are all factual statements correct and adequately supported by citations?”]** 

We thank the Reviewer for clarifying the sample sizes on this discussed paper. We have edited the referenced sentence as follows in light of this information:

*“Genetic variants were chosen by conducting a GWAS within 952 individuals from the LifeLines-DEEP cohort. A fairly lenient p-value threshold of 1x10 ^-5^ (identified from an independent cohort of 445 individuals) was used to define host genetic variants independently associated with functional features of the gut microbiome and explained most variance in the same feature.”*

**5. Page 7 second paragraph. Please add the note that some of the complimentary approaches to inform MR (for example the bivariate finemapping utilized by Richardson et al.) – require individual level data of both the exposure and the outcome, and thus are currently impractical to apply to diseases, as there are not such large scale studies of microbiome on case-control studies.** 

We have added the following sentence to reflect this.

*“As these methods (particularly colocalization and genetic fine mapping) require individual-level and genome-wide information on both the exposure and outcome, these methods will become more feasible with the growing availability of large-scale GWASs of the gut microbiome and other traits.”*

**6. Page 2, last sentence of the second paragraph. I suggest to add a sentence remarking that to solve this ambiguity, large studies are extremely needed as they will likely lead to multiple associated SNPs, which will help MR to better investigate effect of confounders.** 

The Reviewer makes an important point and we have added the following sentence to a paragraph in the limitations section to address this:

*“A greater number of genetic variants robustly associated with features of the gut microbiome will further enable the application of the continuously developing plethora of MR methods that require multiple instruments to investigate the effect of confounding, mediation, pleiotropy and invalidation of MR assumptions.”*
